# Custom Cesium-131 Vicryl Mesh Brachytherapy for Recurrent Anal Cancer: A Report of Two Cases

**DOI:** 10.7759/cureus.70015

**Published:** 2024-09-23

**Authors:** Mark E Bernard, Avinash Bhakta, Dennis A Cheek, Marcus E Randall

**Affiliations:** 1 Radiation Oncology, University of Kentucky, Lexington, USA; 2 Surgery, University of Kentucky, Lexington, USA; 3 Radiation Medicine, University of Kentucky, Lexington, USA

**Keywords:** anal, brachytherapy, cancer, cesium, mesh, progressive, recurrent, vicryl

## Abstract

While the standard of care for anal cancer consists of concurrent chemoradiation, patients with advanced T stages often succumb to local failures. Salvage treatment consists of an abdominoperineal resection (APR). While this is a good surgery to treat the local recurrence, there may be a risk of obtaining a positive margin due to the advanced nature and location of the recurrence. Addressing these high-risk positive margin sites with adjuvant brachytherapy after surgical resection is a good option to deliver a high dose of radiation to the R1 resection site while sparing the adjacent critical organs at risk. Herein, we present a case report of two patients with persistent or recurrent anal cancer who were treated with an APR with placement of a custom Cesium-131 brachytherapy mesh implant.

## Introduction

The standard of care for anal cancer consists of concurrent chemoradiation [1]. While several trials have shown high cure rates with this regimen, those with large primaries have an increased risk of failure [1]. Salvage abdominoperineal resection (APR) is the accepted standard for recurrent anal cancers but can be complicated due to the ability to achieve negative margins [2]. While re-irradiation with concurrent chemotherapy has been shown in the literature, the salvage dose of delivery is often limited given prior radiation therapy [3]. However, low-dose-rate (LDR) brachytherapy provides an excellent option to treat high-risk positive margin sites. With this option, dose escalation is achievable given the short range of radiation exposure which protects the previously irradiated organs. Therefore, we present a custom method of using LDR brachytherapy with a custom Cesium-131 (Cs131) Vicryl mesh in two patients treated with salvage APR for high-risk positive margin sites whose cancers progressed through chemoradiation.

## Case presentation

Case 1

**Figure 1:**
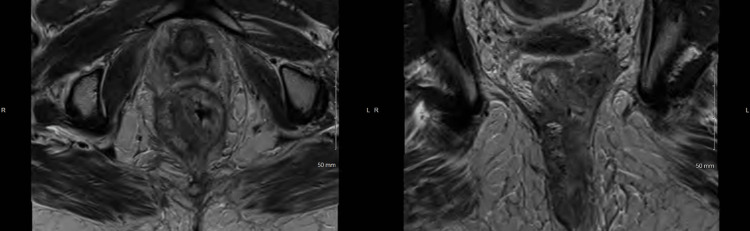
Pelvic MRI showing a locally advanced recurrent anal cancer involving the left pelvic sidewall in the axial (left) and coronal (right) views for case 1

Patient A presented as a 44-year-old female who was initially diagnosed with a T4N1M0 anal squamous cell carcinoma and completed chemoradiation up to 54 Gy at an outside hospital in November of 2018. In March 2019, she experienced anal pain, and a colonoscopy led to a biopsy-proven recurrent anal squamous cell carcinoma. Subsequent pelvic magnetic resonance imaging (MRI) showed a 6.6 cm tumor that was partially circumferential with infiltration in the puborectalis muscle along the posterior left paramedian aspect and a metastatic nodal deposit with extension through the mesorectal fascia and abutment of the underlying pelvic fascia. This can be seen below in Figure 1. She also had a PET/CT scan done which showed no evidence of distant recurrence.

She was taken to the operating room (OR) for an APR with perineal reconstruction and a right vertical rectus abdominis myocutaneous flap. Final pathology confirmed an invasive moderately differentiated keratinizing squamous cell carcinoma of the anus with multiple positive margins involving the circumferential margin, left pelvic side wall, vaginal margin, and sacral margin. One of the nine lymph nodes was positive for malignant disease. The plan was to return to the operating room for management of the myocutaneous and omental flap as well as coccygectomy. Radiation oncology was consulted to see if further radiation could be given to the positive margin site along the left pelvic side wall. The sites and areas of concern were reviewed in a multidisciplinary fashion with colorectal surgery. It was determined to make a custom Cs131 mesh to cover a 7x7 cm site for the left pelvic sidewall margin site to a dose of 50 Gy to a 5 mm depth of the treatment plane. This was done in a multidisciplinary fashion with radiation oncology, colorectal surgery, and radiology. We were given the opportunity to determine the treatment area. Our custom mesh consisted of seven sets of stranded seeds, each containing eight seeds. Relevant calculations have been previously described [4-5]. The total activity was calculated in Au198 and then converted to Cs131 [5].

**Figure 2:**
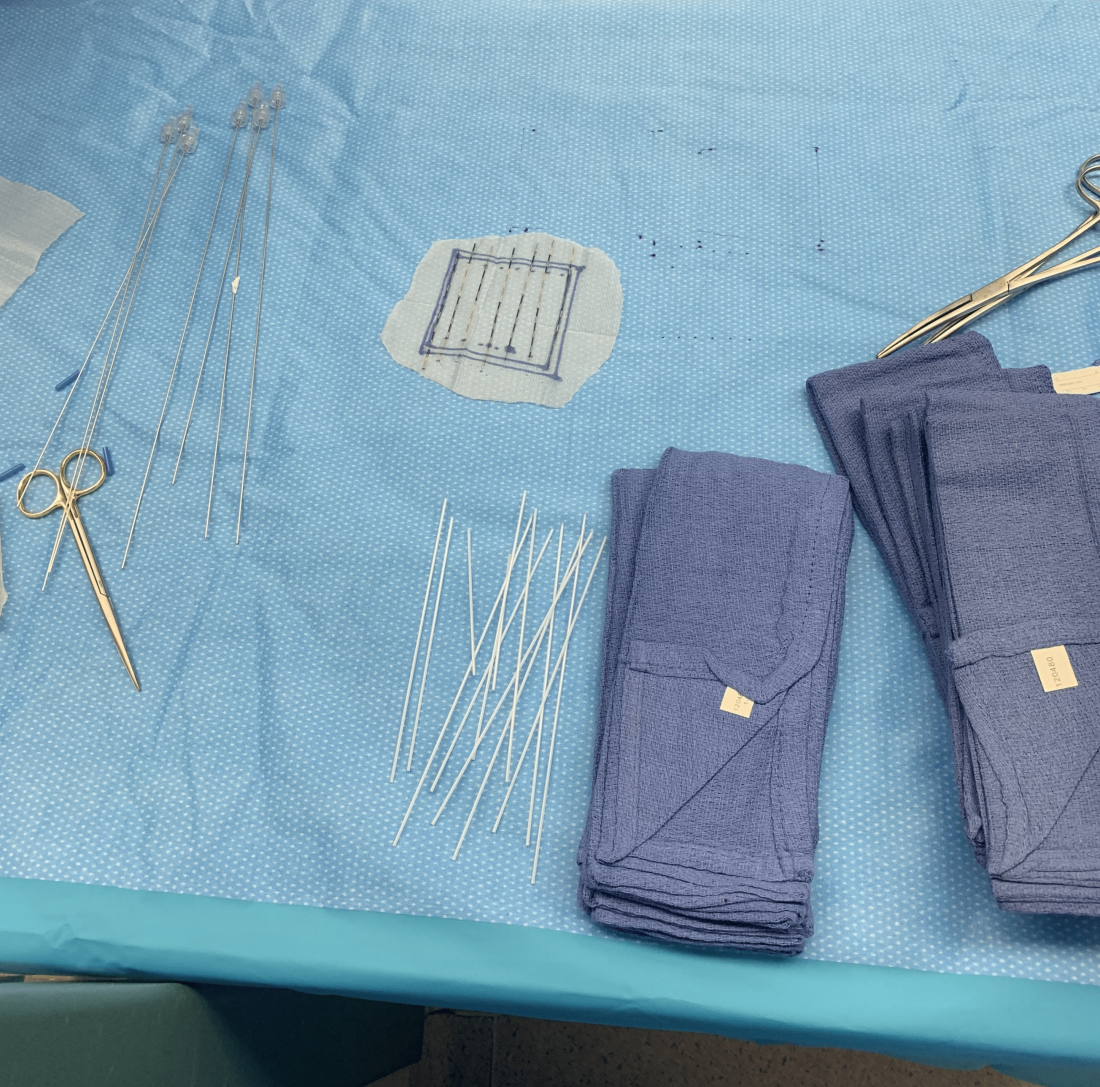
The Cesium-131 stranded seeds woven within a Vicryl mesh for case 1

On the day of the operation, the patient was taken to the operating room for her coccygectomy and flap reconstruction. Once needed, we entered the operating room and created a custom Cs131 Vicryl mesh. We first weaved seven flexiguide needles into the Vicryl mesh which measured 8x8 cm and then removed the stylet. Following this, we placed the Cs131-stranded seeds into each hollow stylet holder which was still woven into the Vicryl mesh. Based on our pre-plan, we wanted the spacing for each strand to be 1 cm for the first three stranded seeds, then 1.5 cm between the next two stranded seeds, and then 1 cm apart for the final three stranded seeds. We then removed the needle holders which left us with a Vicryl mesh with woven stranded Cs131 seeds. Figure 2 shows a picture of our custom mesh with the stranded seeds in place.

**Figure 3:**
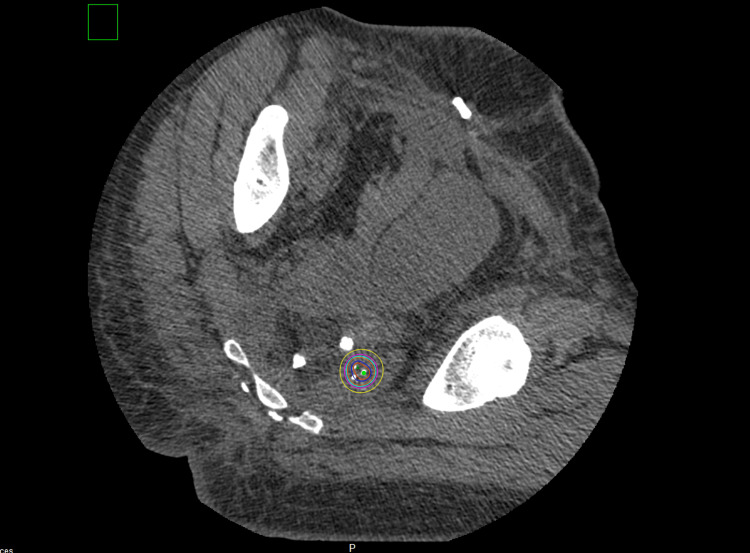
Post-implant CT scan with the Vicryl mesh with Cesium-131 stranded seeds in the expected location for case 1 Red = 200% isodose line (ISL); blue = 150% ISL; orange = 100% ISL; teal = 90% ISL; magenta = 80% ISL; yellow = 60% ISL

At this point, DuraSeal® solution was placed on the custom mesh and a second layer of Vicryl mesh was placed on top and flattened upon the seeds to create a more stable mesh for the seeds to be implanted. Once dried, the custom Cs131 mesh was sutured onto the left pelvic side wall muscle area with no significant air gaps between the mesh and the area of concern. An omental flap was placed over the mesh which protected the adjacent organs. We obtained a post-implant CT scan eight days later. Figure 3 shows the post-implant CT scan with the mesh in the expected location. The mesh covered the high-risk positive margin site at the left pelvic sidewall. The dose delivery location was acceptable. Figure 3 below shows the post-implant CT scan with the Vicryl mesh with Cs131-stranded sees in the expected location.

She had a follow-up with us approximately two months after the completion of the procedure and was recovering well with no radiation-related side effects. She was also seen at approximately three months follow-up with no radiation-related side effects. Unfortunately, she was eventually lost to follow-up.

Case 2

**Figure 4:**
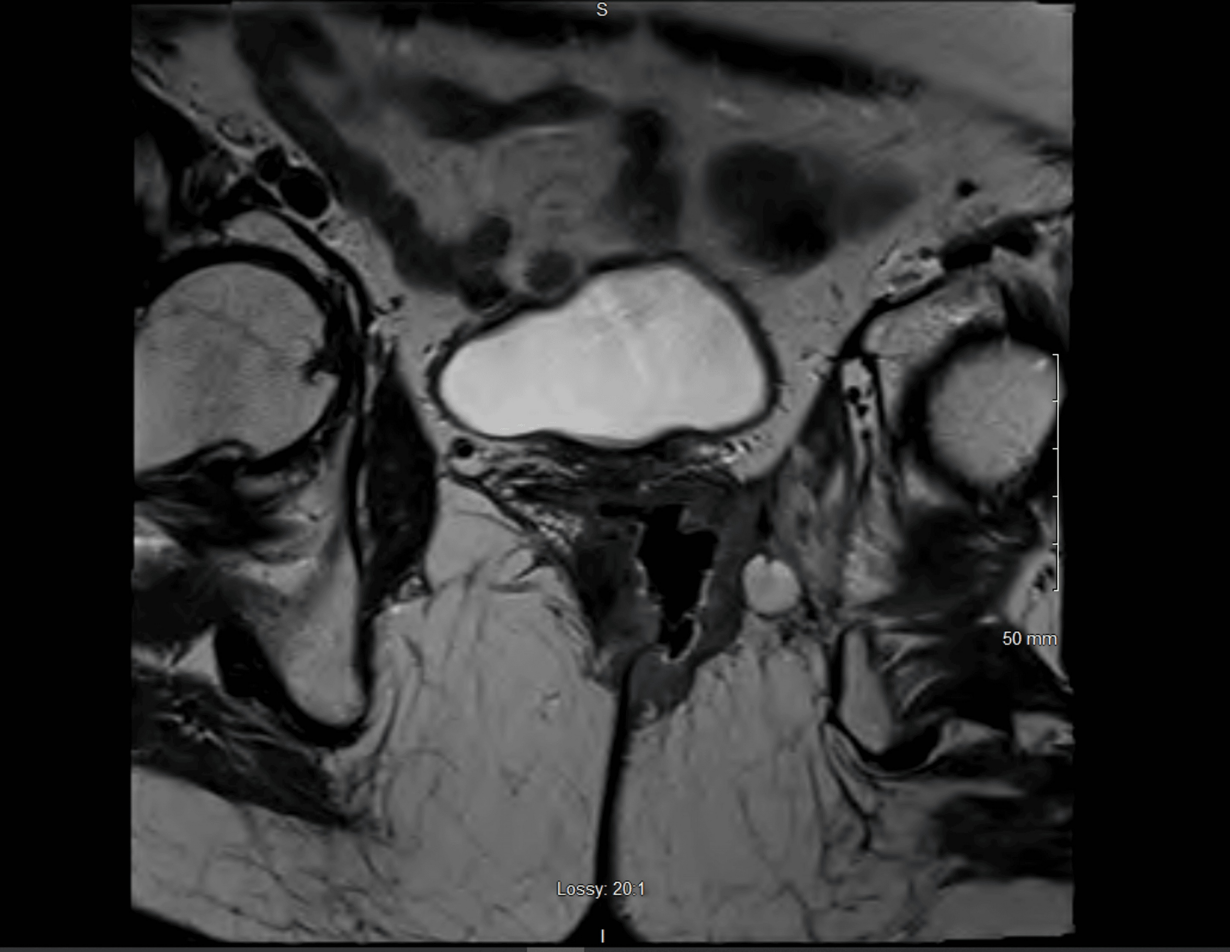
Pelvic MRI showing locally advanced recurrent anal cancer in the axial planes for case 2

Patient B is a 55-year-old female who was diagnosed with anal cancer and was treated with definitive chemoradiation in 2016 and had a complete response on colonoscopy. Unfortunately, she was lost to follow-up but developed increasing rectal pain which led to a biopsy-proven anal squamous cell carcinoma which was circumferential on colonoscopy in 2019. Subsequent MRI showed an infiltrating anal mass with fistulization of the anal canal and vagina anteriorly along with invasion into the puborectalis muscle and left lateral pelvic sidewall. Restaging scans showed no evidence of metastatic disease. Figure 4 shows the pelvic MRI of the mass.

**Figure 5:**
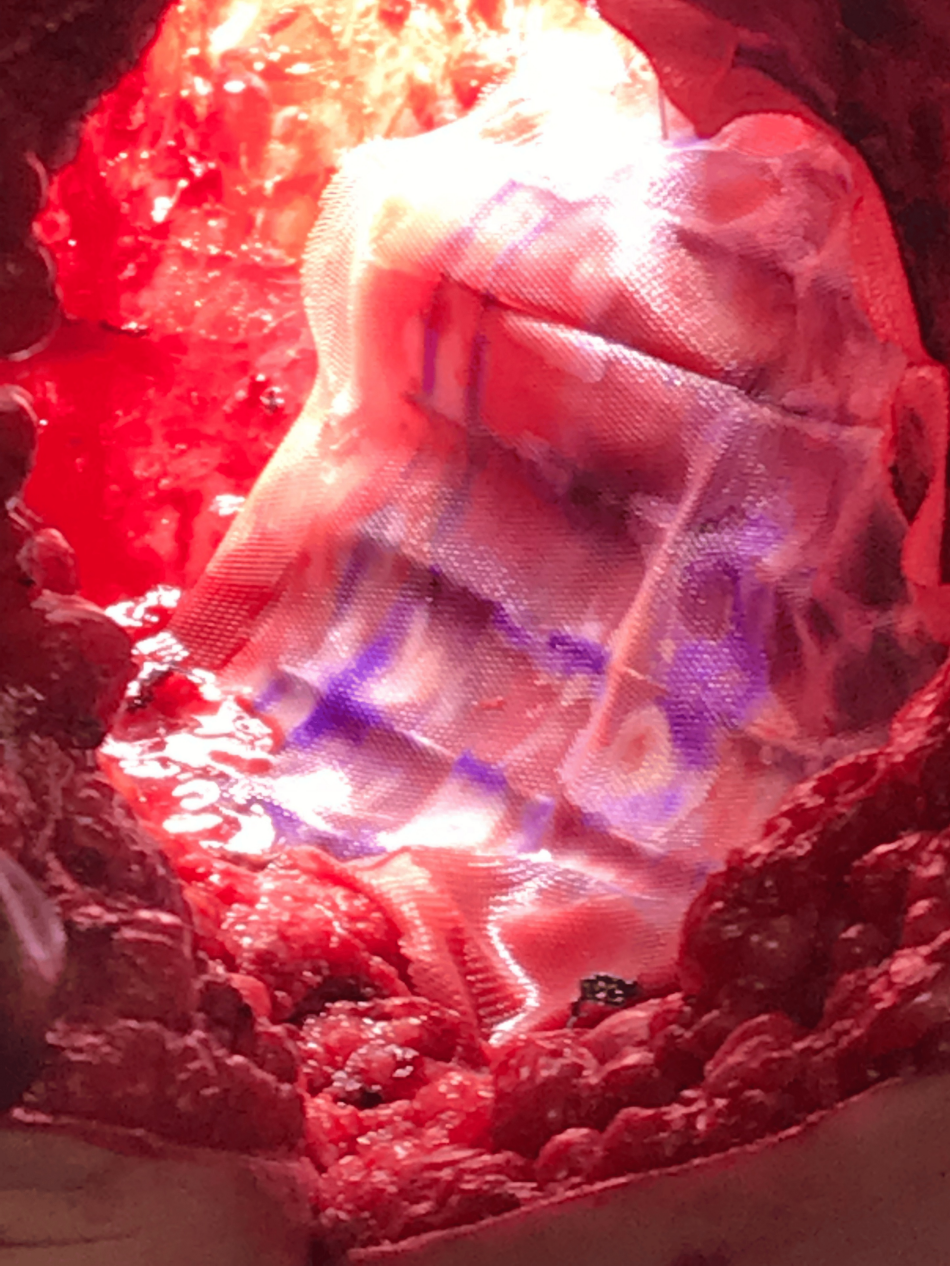
The Cesium-131 Vicryl mesh sutured along the left lateral pelvic side wall before closure for case 2

Her case was discussed in a multidisciplinary fashion with colorectal surgery and there was strong concern for a positive margin along the left sidewall with surgery. Her performance status was 80% upon initial evaluation with radiation oncology. However, she was started on a pre-rehabilitation regimen where her nutritional status was optimized before surgery which can help to improve her performance status which she tolerated well. She was evaluated afterward, and it was decided to perform a pelvic exenteration, omental flap creation, and plastic reconstruction, with the placement of a custom Cs131 Vicryl mesh along the left lateral pelvic wall. Similar to the first patient, a pre-plan was created. On the day of the operation, the patient was taken to the operation for her above-mentioned surgery. A similar protocol was followed, and the custom Cs131 mesh was sutured onto the left obturator muscle area with no significant air gaps between the mesh and the area of concern. An omental flap was eventually placed over the Vicryl mesh. Figure 5 shows the mesh within the left obturator space.

**Figure 6:**
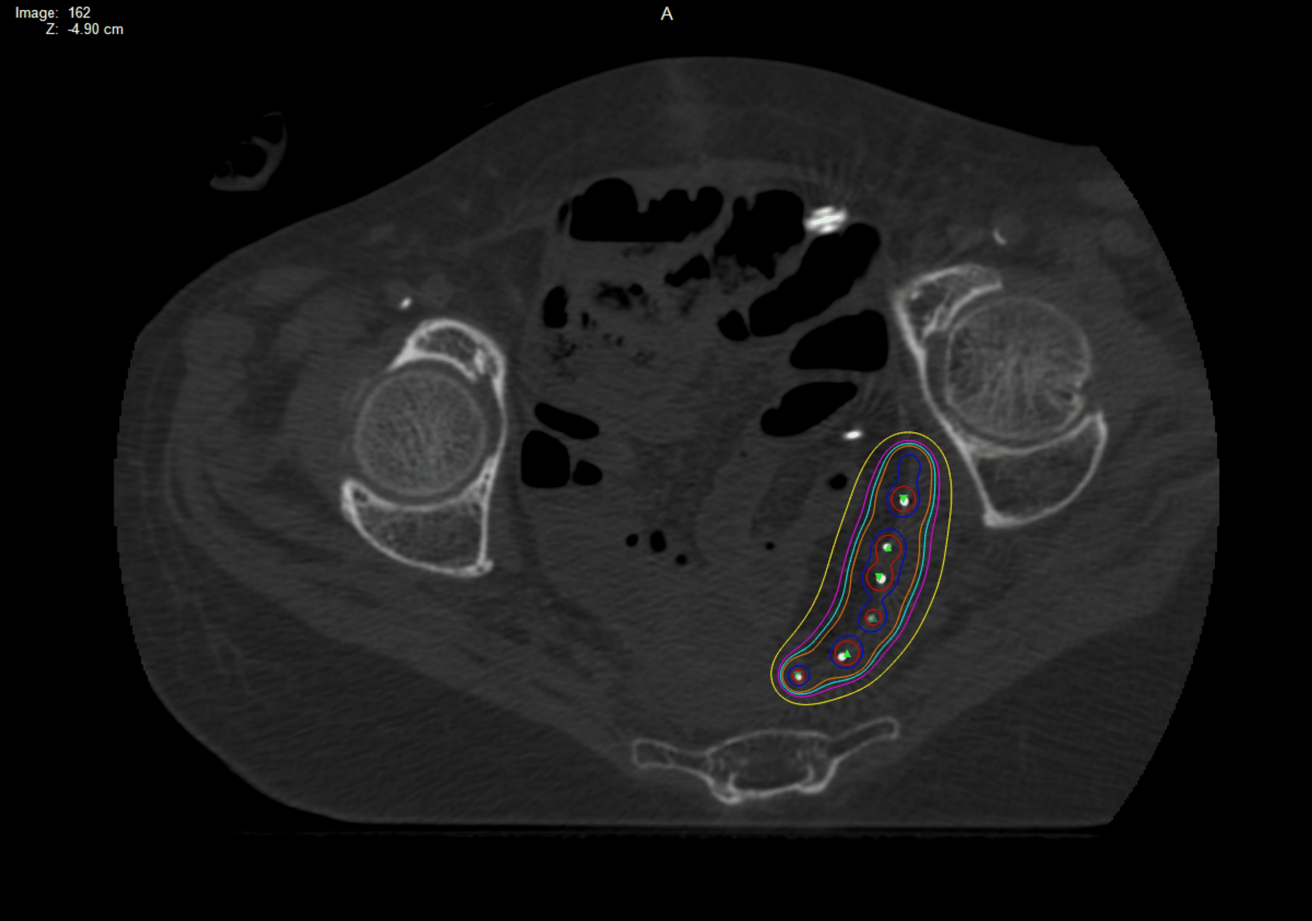
Post-implant CT scan with the Vicryl mesh with Cesium-131 stranded seeds in the expected location for case 2 Red = 200% isodose line (ISL); blue = 150% ISL; orange = 100% ISL; teal = 90% ISL; magenta = 80% ISL; yellow = 60% ISL

There were no immediate complications and the patient was eventually discharged home 11 days later. Her post-implant CT scan shows the mesh within the correct location. The treatment isodose lines covered the majority of the high-risk positive margin sites. The dose delivery location was also acceptable. This is seen in Figure 6.

Unfortunately, she later developed multiple small bowel obstructions, with no clear association with the mesh placement. She was later found to have leukocytosis and malaise and was diagnosed with an abdominal phlegmon. Imaging findings during this time showed no evidence of recurrence. She was also found to have midline wound dehiscence. Interventional radiology was consulted for drainage but elected not to do so since no well-formed abscess was found. She was admitted again into the hospital with a left lower quadrant/perineal abscess and had a drain placed and placed on antibiotics. She sadly developed severe dehydration associated with lactic acidosis and sepsis. During this, she was placed on hospice.

## Discussion

Concurrent chemoradiation has been the standard of care for anal squamous cell carcinomas [1]. There have been high cure rates, but advanced T stages are still associated with a low colostomy-free survival rate of approximately 60% [1]. Salvage APR remains the standard of care for these patients with moderate salvage success rates up to approximately 65% [6]. However, it can be difficult to achieve negative margins and positive margins are associated with inferior outcomes [7]. Therefore, when planning for such an extensive surgery, addressing a negative margin is very important. Several retrospective reviews have looked at radiation techniques in this setting including external beam radiation therapy and intraoperative radiation therapy (IORT). Here, we present our novel method of using LDR brachytherapy by creating a custom Cs131 Vicryl mesh.

One method of achieving a negative margin is through re-irradiation with pre-operative external beam therapy. Unfortunately, this can be limited since most of these patients have received a full course of chemoradiation with doses ranging from approximately 50 to 59.4 Gy.

Osborne et al. reported on 10 patients with recurrent anal cancer who previously received pelvic chemoradiation to at least 30 Gy and received hyperfractionated salvage radiation with either curative (3) or pre-operative (7) intent [3]. The median dose was 39 Gy (range: 27-45 Gy). The three-year overall survival (OS) rate was 60%. Five (71%) of the pre-operative patients received surgery and three (60%) were disease-free at a median of 43 months. Two of the five patients achieved an R0 resection and three had an R1 resection. All three patients treated with definitive re-irradiation alone were alive at a median duration of 84 months. There was only one grade three acute toxicity reported and there were no grade three or higher late morbidity reported. This shows external beam is efficacious, but the lower dose of re-irradiation for the pre-operative group (range: 27-39 Gy) could be associated with the 60% R1 resection rate.

The Mayo Clinic reported on a series of 32 patients with residual or recurrent anal squamous cell carcinoma following treatment with radiation with or without chemotherapy (two patients had radiation alone) [8]. Out of this cohort, 23 patients (72%) had recurrent malignancy. The median re-irradiation dose was 30 Gy (range: 10.8-45 Gy) using a BID fractionation regimen. The five-year OS was 23% with R1 (41% of patients) and R2 (9% of patients) resection margins being associated with inferior survival. There was a 47% grade 3 treatment-related morbidity rate.

The University of Miami reported on 14 patients who received high-dose-rate IORT for 14 patients with locoregionally recurrent anal cancer. A median external beam radiation dose of 15 Gy (range: 15-17 Gy) was given to the high-risk areas of the tumor bed. There were several acute toxicities including wound healing complications, gastrointestinal obstruction, neurogenic bladder, and peripheral neuropathy. While this technique seems feasible, this technology isn’t readily available at every institution.

This case report is unique in that our custom mesh technique can deliver a high dose of radiation while sparing the critical organs given the short distance and is feasible for any radiation oncologist with brachytherapy experience. It has been previously reported on lung, brain, and head and neck cancers [9]. It does not require special technology to be available and allows for multidisciplinary collaboration with surgery and radiology. In both cases, the areas of concern were identified on pre-surgical imaging and within the OR. The mesh could be easily sutured onto the side wall and our lead gloves allowed for low exposure to healthcare providers. We have previously reported on the use of Cs131 for recurrent gynecologic malignancies in the pelvis and had good results using both free-handed methods and template-based methods [5]. We prefer using LDR over a high dose rate (HDR) since LDR may better protect the normal tissue due to the low dose rate also going to the adjacent organs at risk. Also, HDR would require special shielding in the OR, which may not be available at every institution. Last, LDR allows us to create a free-hand custom mesh. Cs131 is our isotope of choice given the 10-day half-life and low energy which leads to less exposure to healthcare providers and caretakers.

However, patients with good performance should be carefully selected for both the surgical procedure and placement of the brachytherapy mesh since complications can arise that may be related to the mesh placement or surgical management of both. A patient's functional status has been shown to correlate with overall survival in the metastatic setting [10]. Even though brachytherapy is a good option for patients with poor performance [11], efforts should be taken to make sure patients who receive the treatment described in this manuscript have an optimal nutritional status, as some patients may have treatment-related morbidity. A good performance status may help to mitigate the detrimental consequences of treatment-related morbidity.

## Conclusions

Our custom LDR Cs131 mesh provides a unique method of addressing high-risk positive margin sites for patients with recurrent anal cancers that have progressed through chemoradiation. It is easily adaptable for any radiation oncologist with adequate brachytherapy experience. It can be performed in the operating room with no need for expensive installations or special shielding. The low half-life and low energy of Cs131 allow for a sufficient dose of radiation while protecting healthcare providers and patient caretakers. The appropriate patients should be selected for this technique, which includes patients with a good performance status who will comply with scheduled follow-ups.
